# Novel *Chlamydia*-like Organisms as Cause of Bovine Abortions, UK

**DOI:** 10.3201/eid1608.091878

**Published:** 2010-08

**Authors:** Nicholas Wheelhouse, Frank Katzer, Frank Wright, David Longbottom

**Affiliations:** Moredun Research Institute, Edinburgh, Scotland, UK (N. Wheelhouse, F. Katzer, D. Longbottom); Scottish Crop Research Institute, Dundee, Scotland, UK (F. Wright)

**Keywords:** Chlamydiales, cattle diseases, abortion, zoonoses, bacteria, United Kingdom, letter

**To the Editor:** Despite the worldwide economic impact of, and welfare issues associated with, infectious bovine abortifacients, as well as potential zoonotic threats to human health, accurate diagnosis of the causes of abortion is uncommon (*1*). This poor diagnosis could be explained in part by the lack of identification of infectious abortifacient agents.

Although *Chlamydophila abortus* is a known etiologic agent of ruminant abortion, several novel species of *Chlamydia*-like organisms have recently emerged as putative ruminant abortifacients. *Waddlia chondrophila* was isolated from the brain and nervous tissue of an aborted bovine fetus in Germany (*2*), and *Parachlamydia acanthamoebae* and other unidentified *Chlamydia*-like species were identified in 18.3% of bovine placenta samples in Switzerland (*3**,**4*). Given the paucity of information about the causes of infectious bovine abortion and the high prevalence of *Chlamydia*-like organisms in the samples from Switzerland, we attempted to determine whether such organisms can be detected in bovine fetal tissues in the United Kingdom.

Pooled tissue samples comprising brain, heart, and/or placenta (depending on availability) were obtained from bovine fetuses submitted for diagnosis to the Scottish Agricultural College Disease Surveillance Centre, Dumfries, Scotland, UK, during 2008. Tissue pools were homogenized by using a Precellys bead mill homogenizer (Bertin Technologies, Ann Arbor, MI, USA), and DNA was extracted by using the Wizard Genomic DNA Purification Kit (Promega, Southampton, UK) according to manufacturer’s instructions. A pan-Chlamydiales PCR for the 16S rDNA sequence was performed by using forward primer 16S FOR2 (5′-CGT GGA TGA GGC ATG CAA GTC GA-3′) and reverse primer 16S REV2 (5′-CAA TCT CTC AAT CCG CCT AGA CGT CTT AG-3′) to generate amplicons of ≈260 bp (*5*). Negative-control reactions contained DNA-free water instead of extracted DNA. PCR products were purified (QIAquick PCR Purification Kit; QIAGEN, Crawley, UK) before direct sequencing by using the PCR primers and dideoxy chain termination/cycle sequencing on an ABI 3730XL DNA sequencer (MWG Operon, Ebersberg, Germany).

After the initial PCR, 22 (26.5%) of the 83 fetal samples tested were Chlamydiales positive. Serologic, bacteriologic, and histopathologic examination of fetal tissues identified no other infectious abortifacient agents in the Chlamydiales-positive samples. Sequence information was successfully obtained for 15 of these 22 samples with forward and reverse primers; sequences ranged from 140 bp to 194 bp (European Molecular Biology Laboratory/GenBank accession nos. GQ919016–GQ919030). These 15 short sequences were carefully aligned to a representative set of 22 similar Chlamydiales 16S rDNA sequences, identified by a BLAST (www.ncbi.nlm.nih.gov/BLAST) similarity search of the European Molecular Biology Laboratory/GenBank database, plus alignment of an outgroup of 7 non-Chlamydiales sequences. A Bayesian phylogenetic tree (Markov Chain Monte Carlo settings: 2 runs of 625,000 generations; burn-in of 125,000 generations; trees sampled every 100 generations) was then estimated with a general time reversible + Γ nucleotide substitution model by using the MrBayes program (*6*) launched from the TOPALi v2 package (*7*).

Despite the short sequence length of the 15 samples, the tree was well resolved with the Chlamydiales sequences and formed 3 clusters (*Chlamydiaceae*, *Rhabdochlamydiaceae*/*Simkaniaceae*, and *Parachlamydiaceae*/*Waddliaceae*/*Criblamydiaceae*) (Figure). Two of these sequence clusters represented 10 and 5 of the samples, whereas no samples were represented in the cluster containing the *Chlamydiaceae*, which includes *C. abortus*. Most (10/15) sequences were found in the cluster containing the *Parachlamydiaceae*. This finding agrees with those of the aborted bovine placenta studies in Switzerland (*3**,**4*) and provides further evidence that *Parachlamydia*-like species may play a substantial role in bovine abortion in mainland Europe and the United Kingdom. Four of the remaining 5 samples clustered with members of the family *Rhabdochlamydiaceae*; the fifth sequence (CLBUK3), although present in the same *Rhabdochlamydiaceae*/*Simkaniaceae* cluster, appeared to be more distinct from other family members.

**Figure:**
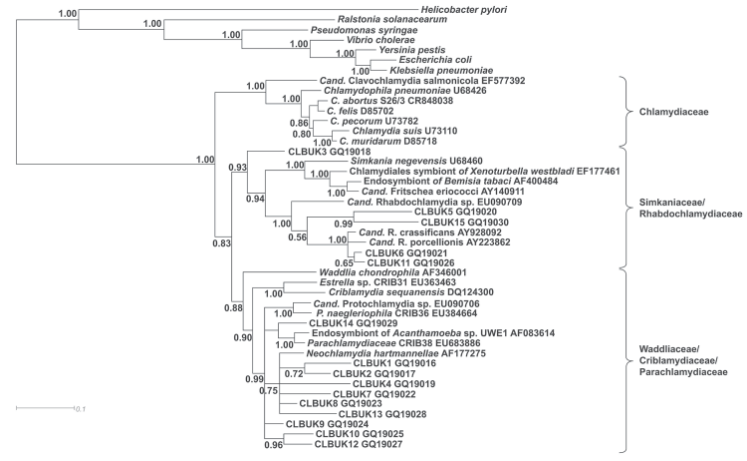
Bayesian phylogenetic tree demonstrating the relationship of 15 isolated organisms from the older chlamydiales samples to known chlamydial species. Cand., *Candidatus*; *R.*, *Rhabdochlamydia*; *P*., *Protochlamydia*.

The identification of these organisms in such a large percentage of the bovine fetal tissue samples tested may indicate a role for these organisms in undiagnosed bovine abortions in the United Kingdom and Europe and may be a zoonotic source of infection for humans. Indeed, considerable evidence supports a role for *Parachlamydia* spp. in human pneumonia, whereas *Rhabdochlamydia* spp. is a suspected cause (*8*). In addition, evidence suggests that *P. acanthamoebae* crosses the human placenta to the unborn fetus (*9*). Also, the presence of both parachlamydial and rhabdochlamydial DNA in the lung secretions of hospitalized premature human neonates recently correlated with increased medical interventions and increased duration of hospital stay (*10*).

We demonstrate the presence of *Parachlamydiaceae* and *Rhabdochlamydiaceae* species in bovine abortions in the United Kingdom. Given the zoonotic potential and the economic and welfare impacts of bovine abortion on the agricultural sector, further studies are required to understand the incidence and pathogenic roles of these organisms in both humans and animals. These studies should include broader molecular epidemiologic studies, as well as detailed histologic/immunohistochemical investigations and organism recovery through culture of infected placental and fetal tissues.
